# Sexually Transmitted Infections Among Active Component Members of the U.S. Armed Forces, 2015–2023

**Published:** 2024-06-20

**Authors:** Armed Forces Health Surveillance Division Defense Health Agency, US

## Abstract

This report summarizes incidence rates and trends of sexually transmitted infections (STIs) from 2015 through 2023 among active component service members of the U.S. Armed Forces. The data compiled for this report are derived from the medical surveillance of chlamydia, gonorrhea, and syphilis as nationally notifiable diseases. Case data for 2 additional STIs, human papilloma virus (HPV) and genital herpes simplex virus (HSV), are also presented. The crude total case rates of chlamydia and gonorrhea initially rose by an average of 6.7% and 9.8% per year, respectively, until 2019. From 2020 onwards, rates steadily declined. By 2023, chlamydia rates had dropped by approximately 39%, while gonorrhea rates had fallen by more than 40% for female, and 19% for male, service members. Initially syphilis increased, on average, 10% annually from 2015 to 2019, then declined in 2020, but resumed its upward trend through 2023, nearly doubling the 2015 rate in 2023. The total crude annual incidence rates of genital HPV and HSV exhibited downward trends in general over the surveillance period, decreasing by 30.7% and 24.7%, respectively. Age- and gender-adjusted case rates for chlamydia, gonorrhea, and syphilis remain elevated within the U.S. Armed Forces compared to the general U.S. population, which may be due to factors that include mandatory STI screening, more complete reporting, incomplete adjustment for age distribution, and inequitable comparisons between the military active duty and general U.S. populations. Social restrictions enacted during the COVID-19 pandemic may have contributed to declines in true case rates and screening coverage.

**What are the new findings?:**

The rates of bacterial STIs have declined since 2020, with the exception of syphilis, which has nearly doubled, increasing by 98% over the 9-year surveillance period. Viral STI rates also declined during the surveillance period. These trends could be influenced partially by changes in screening coverage or behavior associated with the COVID-19 pandemic. Future analyses of screening rates are warranted to assess a true decline in incidence and examine the ongoing rise in syphilis reports.

**What is the impact on readiness and force health protection?:**

To assist service leaders and medical corps in planning and assessing STI prevention and control measures for operational readiness, this report provides an updated epidemiological profile of the most commonly reported STIs. STIs can adversely affect service members’ ability and availability to perform their duties and can result in serious medical sequelae if left untreated. Continued behavioral and educational interventions, focused particularly on those under 25 years of age, are needed to mitigate the risk of STIs among military service members.

## 1 BACKGROUND

In 2022, sexually transmitted infections (STIs) represented one of the highest health care burdens attributable to infectious diseases (other than COVID-19) among active component service members (ACSMs) of the U.S. Armed Forces.^1^ A National Academies of Sciences, Engineering and Medicine committee convened to provide recommendations for prevention and control of STIs in the U.S. concluded that military recruits and active duty service members require focused consideration.^2^ While multiple and interrelated factors influence STI risk within military populations,^3^ the strongest risk factors are age and sex. The military population is young (mean age 26) and predominantly male (85%), so its rates are not directly comparable to the general U.S. population unless adjusted for these demographics.

The Centers for Disease Control and Prevention (CDC) publishes annual summaries of national surveillance data for notifiable diseases including *Chlamydia trachomatis* (chlamydia), *Neisseria gonorrhoeae* (gonorrhea), and *Treponema pallidum* (syphilis) covered under federally-funded control programs.^4^ Although these 3 relatively common bacterial STIs are curable with antibiotics, there is continued concern about the threat of multidrug resistance.^5,6,7^

Common viral STIs in the U.S. also include infections caused by human papillomavirus (HPV) and genital herpes simplex virus (HSV). Studies assessing the National Health and Nutrition Examination Survey (NHANES) data provide prevalence estimates for adolescents and young adults ages 15-24, estimating 1.3 million HSV-2 infections and 9.0 million infected with at least 1 disease-associated HPV type during 2018.^8,9^ Neither HPV nor HSV viral infections are curable with antibiotics; however, suppression of recurrent herpes is attainable using antiviral medication, and a vaccine prevents infection from 4 of the most common HPV serotypes as well as 5 additional cancerous types.^9^

This report presents an epidemiological profile of STIs among U.S. ACSMs from 2015 to 2023, updating previous *MSMR* articles.^10,11^ Data are presented for 5 common STIs: chlamydia, gonorrhea, syphilis, HPV, and HSV.

## 2 METHODS

The surveillance population for this report comprised all ACSMs of the U.S. Army, Navy, Air Force, or Marine Corps who served at any time during the surveillance period of January 1, 2015 to December 31, 2023. Diagnoses of STIs were ascertained from medical administrative data as well as reports of notifiable medical events routinely provided to the Armed Forces Health Surveillance Division and maintained in the Defense Medical Surveillance System (DMSS) for health surveillance. STI cases were also derived from positive laboratory test results recorded in the Health Level 7 (HL7) chemistry and microbiology databases compiled by the Defense Centers for Public Health–Portsmouth (DCPH-P).

For each service member, the number of days in active service was ascertained and then aggregated to a total for all service members for each calendar year. The resultant annual totals were expressed as person-years (p-yrs) of service and used as the denominators for calculating annual incidence rates. Person-time not considered time at risk for each STI—such as the 30 days following each incident chlamydia or gonorrhea infection and all person-time following the first diagnosis, medical event report, or positive laboratory test of HSV, HPV, or syphilis—was excluded. Incidence rates were calculated as incident cases of a given STI per 100,000 p-yrs of active component service, with percent changes in incidence calculated by unrounded rates.

An incident case of chlamydia was defined by either 1) a case-defining diagnosis (**Table 1**) in the first or second diagnostic position of a record of an outpatient or in-theater medical encounter, 2) a confirmed notifiable disease report, or 3) a positive laboratory test (for any specimen source or test type). An incident case of gonorrhea was similarly defined by 1) a case-defining diagnosis in the first or second diagnostic position of an inpatient, outpatient, or in-theater encounter record, 2) a confirmed notifiable disease report, or 3) a positive laboratory test (any specimen source or test type). For both chlamydia and gonorrhea, an individual could be counted as having a subsequent case only if more than 30 days occurred between the dates recorded for each case-defining diagnosis.

An incident case of syphilis was defined by either 1) a qualifying ICD-9 or ICD-10 code in the first, second, or third diagnostic position of a hospitalization, 2) at least 2 outpatient or in-theater encounters within 30 days with a qualifying ICD-9 or ICD-10 code in the first or second position, 3) a confirmed notifiable disease report for any type of syphilis, or 4) a record of a positive polymerase chain reaction or treponemal laboratory test. Stages of syphilis (primary, secondary, late, latent) could not be distinguished because HL7 laboratory data do not allow for stage differentiation, and because a high degree of misclassification is associated with the use of ICD diagnosis codes for stage determination.^12,13^ An individual could be considered an incident case of syphilis only once during the surveillance period; those with evidence of prior syphilis infection were excluded.

Incident cases of genital HSV were identified by either 1) the presence of the requisite ICD-9 or ICD-10 codes in either the first or second diagnostic positions of an outpatient or in-theater encounter record, or 2) a positive laboratory test from a genital specimen source. Antibody tests were excluded because they do not allow distinction between genital and oral infections. Incident cases of genital HPV were similarly identified by either 1) the presence of the requisite ICD-9 or ICD-10 codes in either the first or second diagnostic positions of an outpatient or in-theater encounter record or 2) a positive laboratory test from any specimen source or test type. Outpatient encounters for HPV with evidence of HPV immunization within 7 days before or after the encounter date were excluded, as were outpatient encounters with a procedural or Current Procedural Terminology (CPT) code indicating HPV vaccination, as such encounters were potentially related to vaccination administration. An individual could be counted as an incident case of HSV or HPV only once during the surveillance period. Individuals with diagnoses of HSV or HPV infection before the surveillance period were excluded.

## 3 RESULTS

Between 2015 and 2023, chlamydia was the most commonly detected STI among ACSMs. The number of incident cases of chlamydia exceeded the combined incidence number of the other 4 STIs nearly 2-fold and was 4.5 times the total number of genital HPV infections—the next most frequently identified STI during this period (**Table 2**). The highest rates of chlamydia were observed in the Army (2,290.6 per 100,000 p-yrs) and Marine Corps (2,170.3 per 100,000 p-yrs), while the Navy and Air Force had the highest rates of genital HPV (532.4 and 509.8 per 100,000 p-yrs, respectively).

The total incidence rates of all STIs, excluding syphilis, were higher among female service members, ranging from 1.3 times higher for gonorrhea to 11.2 times higher for genital HPV.

The highest incidence rates for bacterial STIs and genital HSV were concentrated among service members under 25 years of age, while for genital HPV rates were highest among those over 25 years of age. For all STIs, non-Hispanic Black service members, those with a high school education or less, never married, and junior enlisted members had the highest incidence rates.

Patterns of incidence rates over time for each specific STI are described in subsequent subsections.

**Chlamydia**

**Figure 1:**
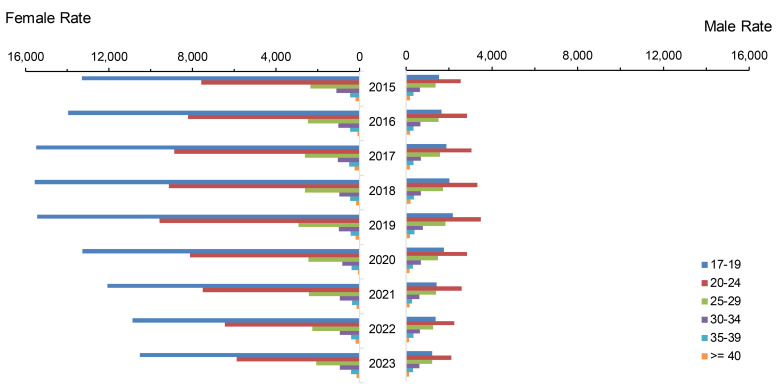
Incidence Rates of *Chlamydia Trachomatis* Infection Among Women and Men by Age Group, Active Component, U.S. Armed Forces, 2015-2023

Annual total chlamydia rates among all ACSMs steadily increased, by an average of 6.7% annually between 2015 and 2019, with rates among both women and men peaking in 2019, at 5,464.3 and 1,886.9 per 100,000 p-yrs, respectively (**Figure 1**). Total chlamydia rates began to decline annually thereafter, with the most pronounced decrease (approximately 21%) observed in 2020. The annual rate of decline in subsequent years averaged approximately 11%.

**Figure 2:**
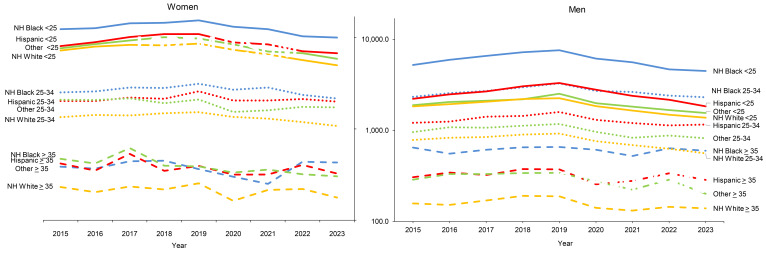
Incidence Rates of *Chlamydia Trachomatis* Infection Among Women and Men, by Age and Racial and Ethnic Groups, Active Component, U.S. Armed Forces, 2015-2023

The younger (under 30 years old) age categories of service members constituted the majority of the overall decline in chlamydia, for both men and women and among all races and ethnicities. The most consistent and largest rate decrease (42.1%) for chlamydia in women was observed among non-Hispanic White service members under age 25, declining from a peak of 8,581.5 cases per 100,000 p-yrs in 2019 to 4,972.4 cases per 100,000 p-yrs in 2023 (**Figure 2**). Among male service members under 25 years of age, the range of declines in incidence rates was between 22% and 44%.

Throughout the 9-year surveillance period, female service members were 3 times more likely to be diagnosed with chlamydia than their male counterparts. The female-to-male ratio of chlamydia infection was highest, at 7 to 9, among those under 20 years of age.

**Gonorrhea**

**Figure 3:**
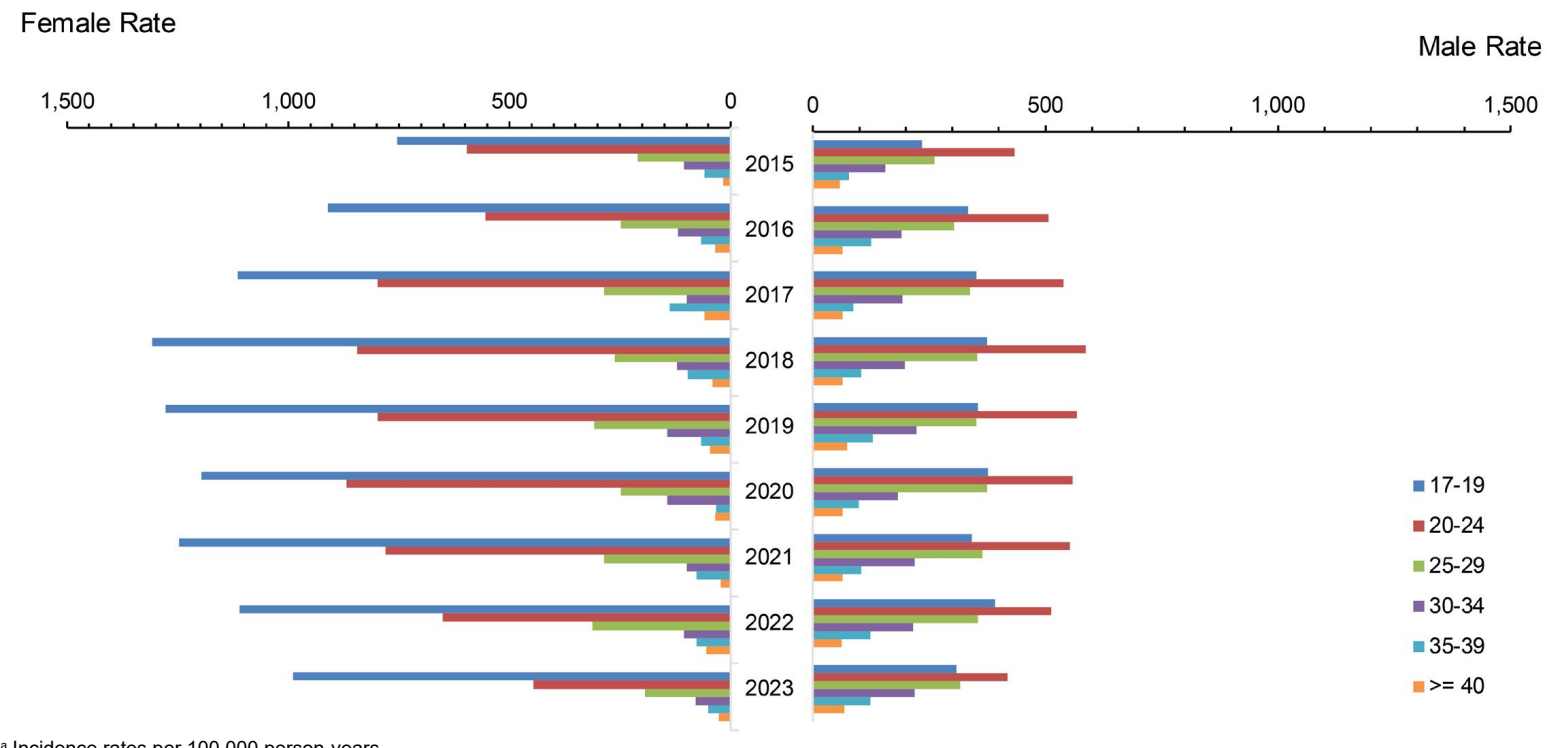
Incidence Rates of Gonorrhea Infection Among Women and Men by Age Group, Active Component, U.S. Armed Forces, 2015-2023

From 2015 to 2019, annual total gonorrhea rates increased by an average of 9.8% per year among all ACSMs, from a low of 264.7 cases per 100,000 p-yrs to the highest recorded rates of 370.60 cases (**Figure 3**). Thereafter, total gonorrhea rates declined on average by 9.3% annually. More substantial declines were observed among women, from a peak of 490.9 cases per 100,000 p-yrs in 2018 to 279.5 in 2023, while rates among men fell from 347.1 per 100,000 p-yrs in 2019 to 280.5 in 2023.

The recent decline in female gonorrhea incidence was primarily driven by rates among service members under age 25 years, although the rate among the youngest female age group, under 20 years, remained elevated in 2023, at 988.7 cases per 100,000 p-yrs, compared to the low of 753.5 from 2015. Among men, the rate decline was most pronounced among those aged 20-24 years, the age group that also had the highest incidence rates. Rates remained relatively stable for age groups over 30, among both women and men.

Throughout the 9-year surveillance period, total incidence rates of gonorrhea in female and male service members were comparable. When disaggregated by age, however, female service members in the youngest age category (under 20 years), were 2.7 to 3.6 times more likely to be diagnosed with gonorrhea than males in the same age range. Among ACSMs older than 25 years, however, women were 10-70% less likely (female-to-male ratio: 0.3-0.9) to be diagnosed with gonorrhea than men. Throughout the surveillance period, gonorrhea rates remained highest among non-Hispanic Black service members, peaking at 1,238.9 per 100,000 p-yrs in 2020, then declining to 833.0 per 100,000 p-yrs in 2023 (data not shown).

**Syphilis**

Syphilis rates steadily rose until 2020, when they declined by 13.2% from the previous year. In 2021, however, syphilis rates resumed their upward trend and in 2023 surpassed all prior year rates from the surveillance period. The crude incidence rate for total cases of syphilis in 2023 (83.3 per 100,000 p-yrs) was almost double the rate observed in 2015 (42.1 per 100,000 p-yrs).

**Figure 4:**
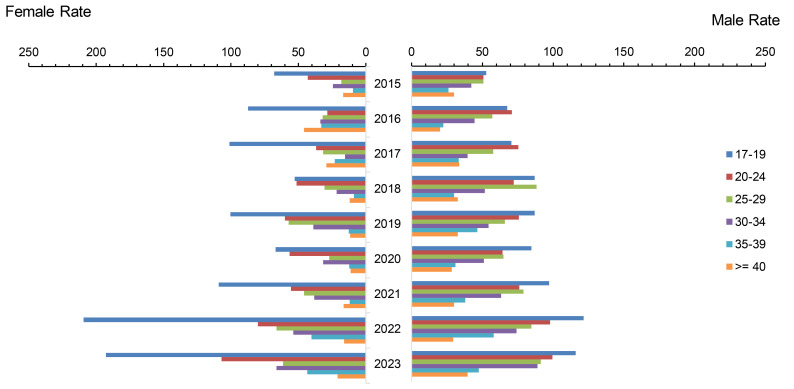
Incidence Rates of Syphilis Infection Among Women and Men by Age Group, Active Component, U.S. Armed Forces, 2015-2023

Female syphilis rates increased by 168.1%, from 30.0 in 2015 to 80.4 per 100,000 p-yrs in 2023, far exceeding—by nearly twice as much—the 89.1% increase in male syphilis rates from 44.3 in 2015 to 83.9 per 100,000 p-yrs in 2023 (**Figure 4**). This notable difference was primarily driven by female service members in the youngest age group, 17-19 years. Syphilis rates among ACSMs ages 17-19 peaked at 209.2 per 100,000 p-yrs in 2022, then decreased by 7.8% to 192.8 cases in 2023, but remained elevated compared to pre-2020 levels. Men ages 17-19 years registered the second highest rate of syphilis, peaking in 2022 at 121.5 cases per 100,000 p-yrs, then declined by 4.5% in 2023, but still elevated more than 2.0 times their lowest levels, in 2015, at 52.6 cases per 100,000 p-yrs.

Syphilis case rates among women in older age groups tended to be lower than the rates among men of corresponding age ranges. Non-Hispanic Black service members consistently accounted for the highest rates of syphilis throughout the surveillance period, with peak rates among women and men under age 25 at 196.7 and 275.0 per 100,000 p-yrs, respectively, and for men ages 25-34 at 260.2 per 100,000 p-yrs in 2023 (data not shown).

**Genital HPV**

**Figure 5:**
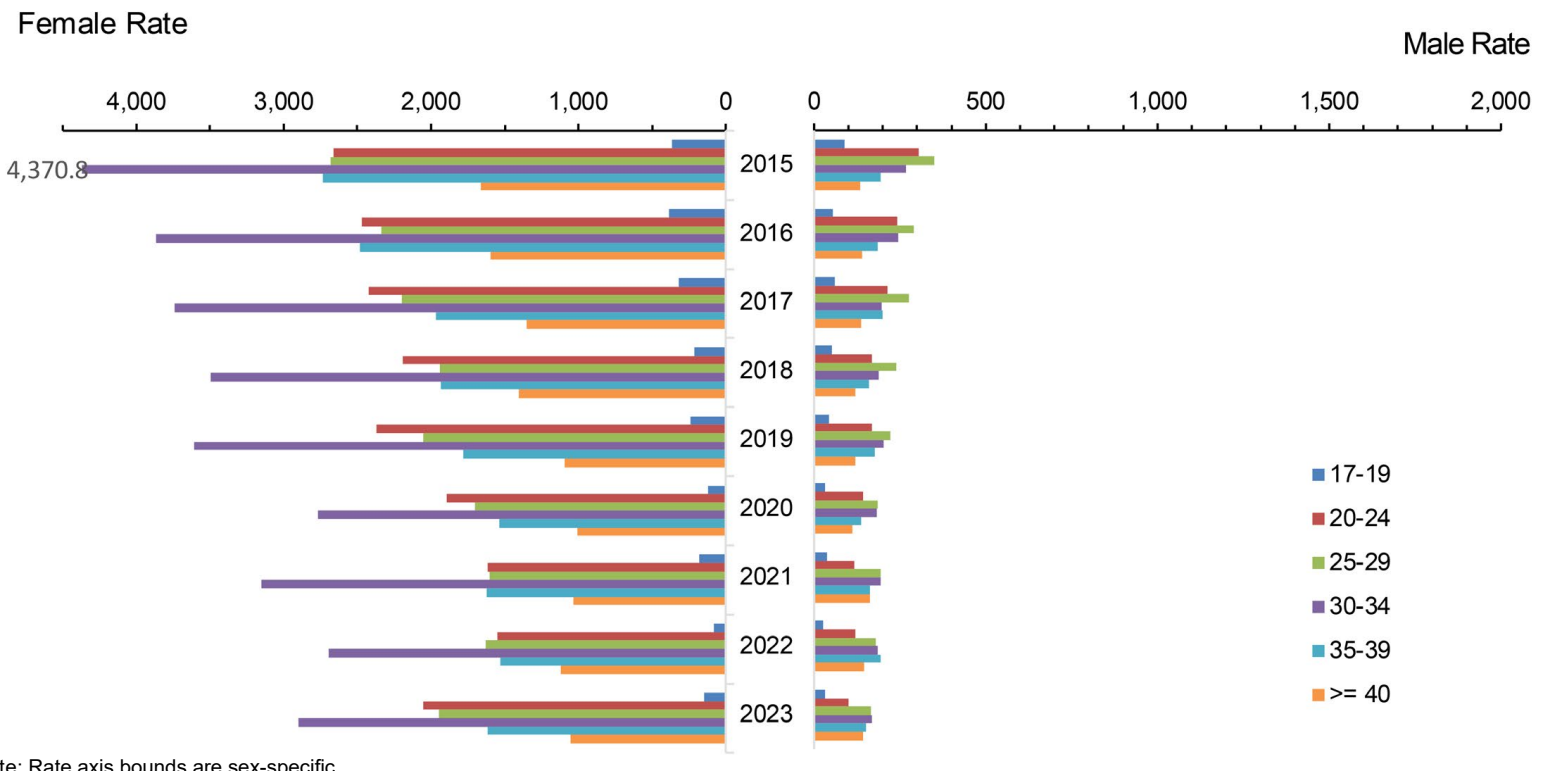
Incidence Rates of Genital HPV Infection Among Women and Men by Age Group, Active Component, U.S. Armed Forces, 2015-2023

The crude annual incidence rates of genital HPV infections among all ACSMs decreased by 30.7% from the start of the surveillance period until its end, but with a more marked absolute decrease among men. The incidence rates of genital HPV infections among male service members decreased from a high of 264.7 cases per 100,000 p-yrs in 2015 to a low of 133.4 in 2023, representing a 49.6% reduction (**Figure 5**). Among female service members, the downward trend continued until 2022, culminating in a 40.0% decline to 1,599.5 cases per 100,000 p-yrs from a high of 2,641.6 in 2015, before increasing in 2023 by 18.6%, to 1,896.8 cases per 100,000 p-yrs. The increased rate in 2023 among women is 28.2% lower than the 2015 peak.

Throughout the surveillance period genital HPV incidence rates displayed marked differences among both sex and age groups. Women ages 30-34 years had the highest rates of genital HPV, as much as 19 times greater than the corresponding rates among men, while women ages 17-19 years had the lowest rates. The HPV rates among the youngest group of women were markedly lower, up to 20 times, than the rates among women in the 30-34 age group.

**Genital HSV**

**Figure 6:**
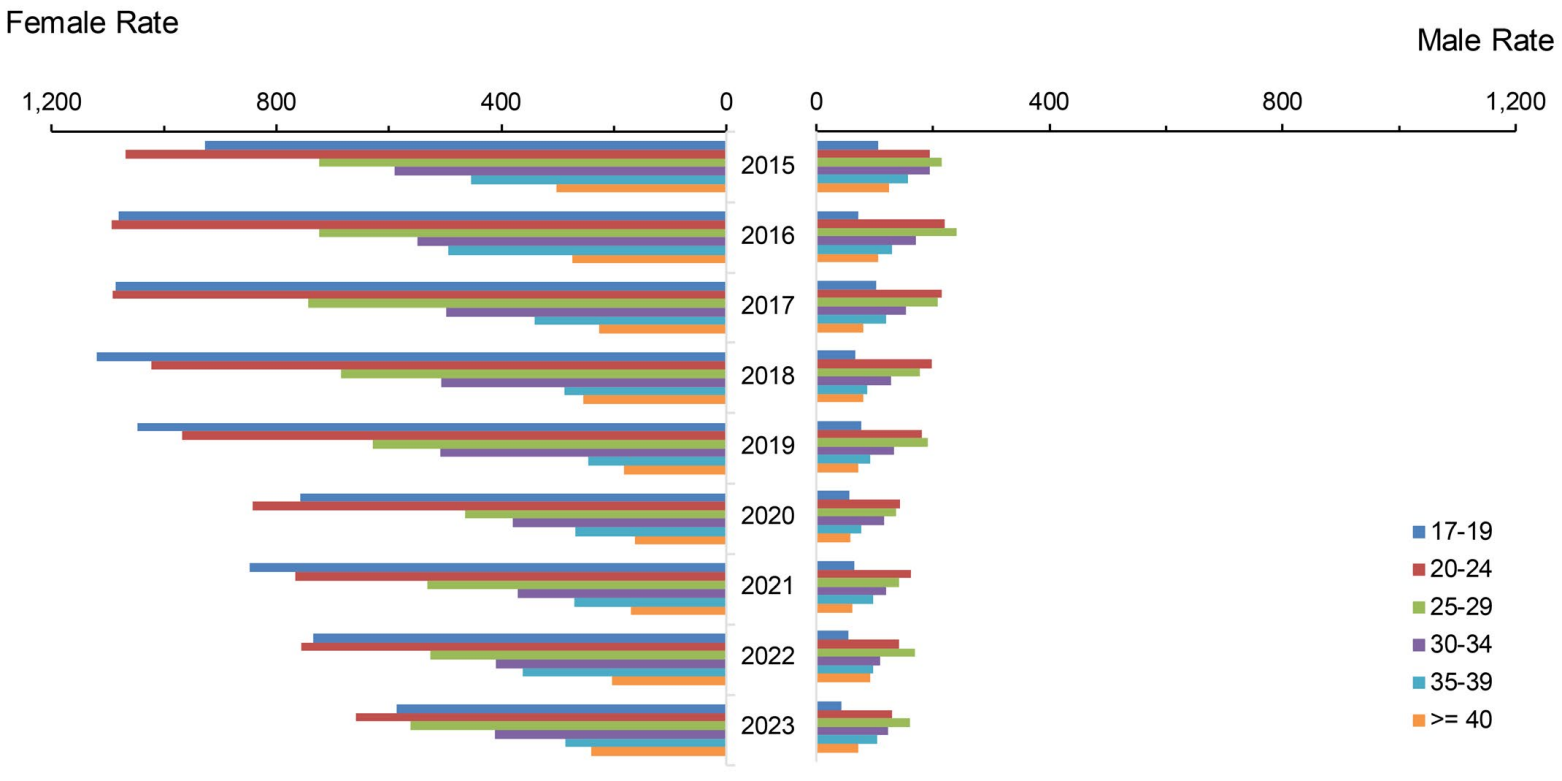
Incidence Rates of Genital HSV Infection Among Women and Men by Age Group, Active Component, U.S. Armed Forces, 2015-2023

From 2020 to 2023 the HSV incidence rate decreased by 24.7% to an average of 194.3 (range: 188.5-199.9) cases per 100,000 p-yrs, after incidence rates fluctuated from 2015 through 2019, with an average of rate at 258.1 (range 36.0–277.4) cases per 100,000 p-yrs. Stratified by sex, HSV rates among female service members declined from a high in 2016 of 786.6 per 100,000 p-yrs to a low of 512.6 per 100,000 p-yrs in 2023 (**Figure 6**). HSV rates for male service members were also highest in 2016 (184.5 per 100,000 p-yrs), then hit their lowest point in 2020 (115.1 per 100,000 p-yrs; 37.7% reduction) before increasing by 5.1% to 121.0 in 2023. On average, female rates were 4 to 5 times higher than corresponding rates among males.

## 4 DISCUSSION

This report provides a surveillance update on 3 nationally notifiable bacterial STIs: chlamydia, gonorrhea, and syphilis; as well as 2 viral STIs: genital HSV and HPV. Chlamydia was the most frequently reported STI, with average incidence rates exceeding those of HPV, the second-most common STI by approximately 2 times. Genital herpes was the third-most detected STI, followed by gonorrhea and syphilis.

Although chlamydia rates were more than 10-fold higher than the rates of gonorrhea, temporal trends in incidence rates for both infections followed a similar pattern. During the initial 5 years of the surveillance period, both chlamydia and gonorrhea showed an upward trend, peaking in 2019, before declining in 2020. Those declines persisted through 2023. An assessment of screening rates may clarify whether this finding constitutes a true decline in incidence, as overall chlamydia and gonorrhea rates in the civilian population continued to increase during this period. The latest CDC report shows that, over the decade spanning 2013 to 2022, chlamydia rates among civilian men increased by nearly 40%, while those among women showed negligible change.^14,15^ Overall rates of gonorrhea more than doubled during the same period, surging by 117% among men and nearly 50% among women.^16^ In absolute terms, the total case rates of chlamydia and gonorrhea among service members were higher than in the general population, for both men and women. In 2022, the chlamydia rates in male and female service members were approximately 3.4 and 5.9 times higher, respectively, than comparable civilian counterpart rates, while the same rates for gonorrhea were 1.5 to 2.6 times higher.

These rate comparisons should, however, be interpreted with an understanding of the unique surveillance methods for each population, as well as their differences in screening access and use. The U.S. military represents a ‘healthy worker’ population with no-cost access to complete preventive and primary care, for maintaining a military ready force.^17^ The electronic health records generated by the Military Health System (MHS) also enable more complete disease burden capture for notifiable disease reporting.

Laboratory and medical encounter data from service members in 2022 supplemented chlamydia case rates, as those cases had no medical event report and would have been unidentifiable without supplemental electronic health record data. Routine surveillance reports do not assess anatomic sites from gonorrhea case reports and laboratory records, which could provide more comprehensive understanding of extragenital infections in high-risk populations.

National guidelines recommend gonorrhea screening, including pharyngeal or rectal testing at least annually for both MSM and HIV-positive patients. Extragenital gonorrhea screening may be considered for women on the basis of reported sexual behaviors and exposure.^18^ Despite these recommendations, extragenital screening for high-risk civilian and military populations is under-utilized.^19^ A recent assessment of extragenital STI screening by primary care physicians for HIV-positive Airmen found that approximately one-third of patients had undetected STIs, the majority due to extragenital infections of the rectum and pharynx.^19^

The trend in syphilis rates reveals a pattern that differs from the other 2 bacterial STIs, reflecting differing epidemiological factors and clinical dynamics. Despite a brief decline in 2020, syphilis rates essentially doubled (98%) over the 9-year period, peaking in 2023. This syphilis surge among the military population exceeds the 80% increase in cases in the U.S. general population from 2018 to 2022, at the highest levels since the 1950s, according to the CDC.^4^

A male-to-female syphilis rate ratio greater than 1.0 persisted, except among women ages 17 to 19 years, throughout the surveillance period. This relative increase in syphilis rates for female service members in the youngest age group could indicate either improved accession screening or a true increase in rates. This finding is reflected in national surveillance reports.

Although civilian rates of primary and secondary syphilis are lower among women, their incidence rates more than doubled from 2016 to 2020.^20^ This evidence reinforces U.S. Preventive Services Task Force recommendations for early syphilis infection screening in all pregnant women.^21^ This recommendation may be particularly relevant for female service members, as the majority of them are largely of childbearing age.

In 2020 all STI rates notably decreased. As noted in CDC national surveillance reports, the COVID-19 pandemic likely contributed to changes in STI screening coverage; thus, incidence rates for that period should be interpreted with caution.^14^ Future analyses of ACSM STI screening practices may help reveal true incidence rate declines, particularly for STIs more commonly associated with asymptomatic infection.

No sexual risk behavior data were available, but prior surveys of military personnel indicate increased behaviors of possible concern. The 2018 Department of Defense Health Related Behaviors Survey (HRBS) documented 19.3% of active component respondents reporting 2 or more sexual partners within the past year, with 34.9% reporting sex without condom use with a new partner in the past year—percentages almost double those in the 2011 survey.^22^

This report has several limitations that should be considered. First, STI diagnoses can be incorrectly coded. For example, STI-specific “rule out” diagnoses or vaccinations (e.g., HPV vaccination) may be reported with STI-specific diagnostic codes, which would result in STI incidence overestimation. Cases of syphilis, genital HSV, and genital HPV infections based solely upon laboratory test results are considered “suspect” because laboratory results cannot distinguish between active and chronic infections. Because incident cases of those 3 STIs were identified based upon a first qualifying encounter or laboratory result, it is likely most cases were acute and not chronic.

STI cases coded in the medical record using symptom codes (e.g., urethritis) rather than STI-specific codes may not be captured. In addition, the counts of STI diagnoses reported here may underestimate actual diagnoses because some service members may have been diagnosed and treated by non-reimbursed, non-military care providers (e.g., county health departments, family planning centers) or in deployed settings (e.g., overseas training exercises, combat operations, aboard ships). Laboratory tests in the private sector care or in a shipboard facility, battalion aid station, or in-theater facility were not captured in this analysis.

For some STIs, detection of prevalent infection may occur long after initial infection. Changes in incidence rates may reflect, at least in part, temporal changes in case detection, including more aggressive screening. The lack of standard service and installation practices for STI screening, testing, treatment, and reporting complicates interpretations of detected differences between services, military and demographic subgroups, as well as locations. Standard STI screening, testing, treatment, and reporting throughout the services, and consistent adherence, can improve detection and characterization of STI-related health threats. Continued behavioral risk-reduction interventions are still required to counter STIs among military service members.

## Figures and Tables

**Table 1:** ICD-9 and ICD-10 Diagnostic Codes Used to Identify STI Cases in Electronic Health Care Records

STI	ICD-9^a^	ICD-10^a^
HPV	078.11, 079.4, 795.05, 795.09, 795.15, 795.19, 796.75, 796.75	A63.0, R85.81, R85.82, R87.81, R87.810, R87.811, R87.82, R87.820, R87.821, B97.7
Chlamydia	099.41, 099.5*	A.56.*
Genital HSV	054.1*	A60.*
Gonorrhea	098.*	A54.*
Syphilis	091.*, 092.*, 093.*-0.96*, 097.0, 097.1, 097.9	A51.* (excluding A51.31), A52.*, A53.0, A53.9

Abbreviations: ICD-9, International Classification of Diseases, 9th Revision; ICD-10, International Classification of Diseases, 10th Revision; STI, sexually transmitted infection; HPV, human papillomavirus; HSV, herpes simplex virus.

^a^ An asterisk (*) indicates that any subsequent digit or character is included.

**Table 2:** Incident Counts and Incident Rates of STIs, Active Component, U.S. Armed Forces, 2015-2023

	Chlamydia		Gonorrhea		Syphilis		Genital HSV		Genital HPV	
	No.	Rate^a^	No.	Rate^a^	No.	Rate^a^	No.	Rate^a^	No.	Rate^a^
Total	234,162	2,001.6	39,082	333.6	7,068	60.4	26,500	229.5	51,994	459.7
Sex										
Male	146,952	1,507.2	30,994	317.6	6,136	63.0	14,175	146.7	17,249	180.0
Female	87,210	4,475.8	8,088	413.7	932	47.7	12,325	654.8	34,745	2,009.9
Age group, y										
<20	32,246	3,867.5	4,062	485.9	755	90.3	1,904	228.0	671	80.3
20-24	136,357	3,667.5	20,602	552.7	2,704	72.6	11,137	300.8	18,447	499.7
25-29	45,209	1,659.6	8,799	322.6	1,789	65.7	6,919	257.2	13,762	520.2
30-34	13,543	719.5	3,488	185.2	1,002	53.3	3,521	190.9	11,326	640.4
35-39	4,852	351.0	1,426	103.1	481	34.9	1,866	138.9	4,854	380.3
>=40	1,955	168.8	705	60.9	337	29.2	1,153	102.2	2,934	268.5
Race and ethnicity										
White, non-Hispanic	87,605	1,352.0	10,158	156.6	2,178	33.6	11,385	177.6	23,970	381.4
Black, non-Hispanic	77,034	4,098.2	19,796	1,050.5	2,535	135.0	8,009	436.6	11,664	648.3
Hispanic	46,278	2,369.0	5,697	291.1	1,549	79.3	4,638	240.2	9,548	505.1
Other/unknown	23,245	1,677.5	3,431	247.3	806	58.2	2,468	180.1	6,812	509.6
Education level										
High school or less	202,817	2,738.1	32,721	440.9	5,197	70.1	18,707	254.8	30,827	424.4
Some college	14,372	1,010.8	2,777	195.2	733	51.6	3,179	229.7	7,506	566.8
Bachelor's or advanced degree	14,582	557.8	3,173	121.3	1,020	39.1	4,205	163.9	12,378	500.0
Other/unknown	2,391	936.0	411	160.8	118	46.2	409	161.9	1,283	518.2
Marital status										
Single, never married	165,463	3,248.3	26,605	521.1	4,533	88.9	14,104	278.5	25,101	501.0
Married	54,664	904.6	10,205	168.8	2,104	34.9	9,686	163.0	20,788	358.6
Other/unknown	14,035	2,498.5	2,272	403.8	431	76.8	2,710	503.6	6,105	1,211.4
Service										
Army	96,752	2,290.6	18,946	447.9	2,580	61.1	11,309	271.6	18,004	439.7
Navy	56,215	1,896.8	9,671	325.9	2,641	89.2	6,392	218.6	15,222	532.4
Air Force	45,876	1,591.0	5,892	204.1	1,194	41.4	5,931	208.8	14,044	509.8
Marine Corps	35,319	2,170.3	4,573	280.6	653	40.1	2,868	177.5	4,724	294.8
Rank/grade										
Junior enlisted (E1-E4)	174,629	3,487.4	27,080	539.5	4,487	89.5	14,242	285.4	23,599	474.9
Senior enlisted (E5-E9)	49,930	1,084.2	10,081	218.7	2,029	44.1	9,257	205.6	19,441	447.0
Junior officer (O1-O3)	8,186	700.8	1,457	124.7	374	32.0	2,118	183.4	6,486	574.7
Senior officer (O4-O10)	842	112.4	326	43.5	133	17.8	628	85.5	1,984	280.8
Warrant officer (W01-W05)	575	341.7	138	82.0	45	26.8	255	156.4	484	307.7
Military occupation										
Combat-specific^b^	27,528	1,712.4	4,821	299.5	606	37.7	2,702	169.4	3,669	232.2
Motor transport	11,230	3,262.4	2,148	622.7	416	120.8	994	291.8	1,969	585.4
Pilot/air crew	2,348	554.0	316	74.5	91	21.5	504	120.5	1,227	300.3
Repair/engineering	65,690	1,907.4	10,645	308.7	1,628	47.3	6,917	203.1	12,398	369.6
Communications/intelligence	57,917	2,306.1	10,725	426.4	1,627	64.8	7,229	293.6	14,708	615.9
Health care	17,551	1,733.5	2,909	287.0	594	58.7	2,925	294.5	7,907	830.1
Other	51,898	2,203.8	7,518	318.8	2,106	8.9	5,229	224.4	10,116	441.7

Abbreviations: STIs, sexually transmitted infections; HSV, herpes simplex virus; HPV, human papillomavirus; No., number; y, years.

^a^ Incidence rate per 100,000 person-years.

^b^ Infantry/artillery/combat engineering/armor.

**Figure 7:**
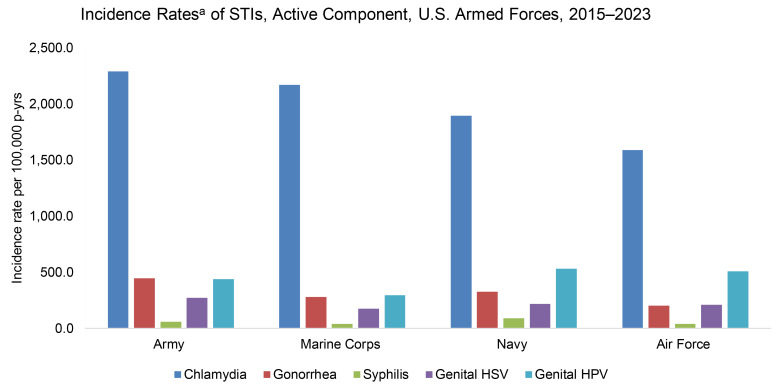
Incidence Rates of STIs, Active Component, U.S. Armed Forces, 2015-2023
